# ELK4-mediated lncRNA SNHG22 promotes gastric cancer progression through interacting with EZH2 and regulating miR-200c-3p/Notch1 axis

**DOI:** 10.1038/s41419-021-04228-z

**Published:** 2021-10-18

**Authors:** Xiaqiong Mao, Tao Ji, Aiguo Liu, Yunqi Weng

**Affiliations:** 1 Department of Gastroenterology, The First Affiliated Hospital of Nanjing Medical University, Nanjing, China; 2 Department of Emergency, The Affiliated Hospital of Qingdao University, Qingdao, China

**Keywords:** Gastric cancer, Long non-coding RNAs

## Abstract

Long non-coding RNAs (lncRNAs) play important regulatory roles in the initiation and progression of various cancers. However, the biological roles and the potential mechanisms of lncRNAs in gastric cancers remain unclear. Here, we report that the expression of lncRNA SNHG22 (small nucleolar RNA host gene 22) was significantly increased in GC (Gastric Cancer) tissues and cells, which confers poor prognosis of patients. Knockdown of SNHG22 inhibited the proliferation and invasion ability of GC cells. Moreover, we identified that the transcriptional factor, ELK4 (ETS transcription factor ELK4), could promote SNHG22 expression in GC cells. In addition, using RNA pull-down followed MS assay, we found that SNHG22 directly bound to EZH2 (enhancer of zeste 2 polycomb repressive complex 2 subunit) to suppress the expression of tumor suppressor genes. At the same time, SNHG22 sponged miR-200c-3p to increase Notch1 (notch receptor 1) expression. Taken together, our findings demonstrated the role of SNHG22 on promoting proliferation and invasion of GC cells. And we revealed a new regulatory mechanism of SNHG22 in GC cells. SNHG22 is a promising lncRNA biomarker for diagnosis and prognosis and a potential target for GC treatment.

## Introduction

GC is one of the most frequently and malignant cancer and the third cause of death of cancer patients [1]. Despite of the aggressive treatment including surgical resection and chemotherapy, the five-year net survival rate of GC patients is still poor, less than 40% [2]. The aggressive phenotypes of GC cells, like enforced proliferation and invasion, is the main reason for the poor prognosis of GC cells [3, 4]. Long noncoding RNAs (lncRNAs) are a class of RNAs molecules that comprise more than 200 nucleotides in length [5–7]. And, lncRNAs have been reported to be dysregulated in cancer tissue compared with adjacent normal tissues, indicating the special function of lncRNAs on regulating tumorigenesis and progression [8, 9].

Many lncRNAs have been reported to be involved in mediating GC progression [10]. SNHG22 is a newly discovered lncRNA, which is translated from *SNHG22* genes located on chromosome 18q21.1. SNHG22 was proved to be overexpressed in epithelial ovarian carcinoma and acts as an oncogene promoting tumor progression [11]. However, the molecular mechanism of SNHG22 in GC cells is not clearly discovered.

In this study, we showed that SNHG22 was overexpressed in tumors of GC patients and it was correlated with advanced pathological phenotype and poor survival. ELK4 transcriptionally elevating SNHG22 expression. SNHG22 promoted GC progression in vitro *and* in vivo by recruiting EZH2 and sponging miR-200c-3p. Our study provided a potential new target for GC treatment.

## Materials and methods

### Patients samples

A total of 60 GC cases who had surgically proven primary GC were obtained from the Affiliated Hospital of Qingdao University. This study was approved by the Ethics Committee on Human Research of the Affiliated Hospital of Qingdao University, and written informed consent was received from all patients. All GC and paired normal tissue samples were collected during surgery and frozen immediately in liquid nitrogen for further total RNA or protein extraction.

### Cell culture

The GSE-1, BGC-823, SGC-7901, MGC-803, AGS, MKN-45, NUGC-3 and HEK 293 T cell lines were purchased from Type Culture Collection of the Chinese Academy of Sciences (Shanghai, China). The cells were cultured in Dulbecco’s modified Eagle’s medium (DMEM) supplementary with 10% FBS, 100 μg/ml streptomycin and 100 U/ml penicillin. All cells were maintained at 37 °C in a humidified atmosphere of 5% CO2.

### RNA preparation and quantitative real-time PCR (qRTP-CR)

Total RNA extraction from GC cells or clinical samples was performed using the TRIzol reagent (Invitrogen, USA) as previously described [12]. For SNHG22 distribution analysis, PARISTM Kit (Thermo Fisher, USA) was used to extract the nuclear and cytoplasmic RNA separately as previously described. Quantitative RT-PCR was carried out using SYBR Green PCR Master Mix (Vazyme) with an ABI Prism 7900 Sequence detection system (Applied Biosystems, Canada). U6 and beta-actin were used as endogenous controls. The primers are shown in Supplementary Table 1.

### Protein extraction and western blot

Protein extraction from cells and tumor tissues and western blot analysis were performed as described as previously described [12]. Antibodies against ELK4 (abcam, ab86002), EZH2 (abcam, ab191250), H3K9me1 (abcam, ab16989), H3K9me2 (abcam, ab1220), H3K9me3 (abcam, ab8898), H3K27me2 (abcam, ab24684), H3K27me3 (abcam, ab6002), H3 (abcam, ab1791), E-cadherin (CST, 14472), EAF2 (CST, 14159), ADRB2 (abcam, ab182136), rap1GAP (abcam, ab32373), RUNX3 (abcam, ab224641), GAPDH (CST, 5174) and Notch1 (CST, 3608) were used.

### Plasmids and siRNA transfection and lentiviral transduction

Lentivirus carrying sh-SNHG22 or sh-ctrl was packaged in human embryonic kidney 293 T cells using the lentiviral packaging kit purchased from Genechem (Shanghai, China). Stable cell lines were established by infecting BGC-823 and MGC-803 cells with lentivirus followed by puromycin selection. siRNA targeting ELK4, miR-200c-3p mimics and anti-miR-200c-3p were designed and synthesized by RiboBio (Guangzhou, China).

### RNA fluorescence in situ hybridization (FISH)

Cy5-labeled specific probe to SNHG22 was purchased from RiboBio and a FISH kit (RiboBio) was used to detect the signals according to the manufacturer’s instruction.

### Cell counting kit-8 (CCK-8) assay

To measure the viability of GC cells, 3 × 10^3^ cells were seeded into 96-well plates, and the CCK-8 solution (Dojindo, KMJ, Japan) was added to each well at the indicated time. After a 1 h incubation at 37 °C, absorbance was detected (OD value) at a wavelength of 450 nm.

### Colony formation assay

To measure the viability of GC cells, 1000 cells were seeded into 6-well plates and cultured for approximately 2 weeks until colony formation was observed.

### Transwell assay

Transwell invasion assay was performed using a 6.5-mm diameter Transwell chamber with 8-μm pore polycarbonate membrane insert (Corning). Matrigel (Corning) was used to cover the upper chambers. 2000 transfected or control GC cells were plated on the upper chambers. 12 h later, cells in the chambers were fixed by 4% paraformaldehyde, stained by crystal violet and counted.

### In vitro 3D migration assay

For 3D migration assays, 20 μl GC cell suspension (containing 2000 cells) was placed onto the lid of a 6-cm dish. The lids were inverted for 2 days to obtain a cellular aggregate. The cellular aggregates were then implanted into three-dimensional collagen I gels (PureCol, Inamed, Fremont, CA, USA). The migration of GC cells was monitored using a Leica DMI3000B microscope system at indicated time.

### Dual luciferase reporter assay

To confirm that SNHG22 could sponge miR-200C-3p, HEK-293T cells were co-transfected with the mixture of luciferase reporter vectors (pmirGLO) containing SNHG22-miR-200c-3p binding sequences or mutant sequences and miRNA mimics (20 nM) to examine the interaction between SNHG22 and miR-200c-3p. A dual luciferase reporter assay system (Promega, Madison, WI, USA) was employed to measure the luciferase activity according to the manufacturer’s protocol.

To explore the transcriptional regulation of ELK4 on SNHG22, HEK-293T cells were co-transfected with luciferase reporter comprising wild type or mutant SNHG22 promoter region and empty vector or ELK4 plasmid (Genechem). A dual luciferase reporter assay system (Promega, Madison, WI, USA) was employed to measure the luciferase activity according to the manufacturer’s protocol.

### Chromatin immunoprecipitation (ChIP)

The Simple Chip Enzymatic Chromatin IP kit (CST, USA) was used to identify the regulation of ELK4 on SNHG22. Briefly, BGC-823 and MGC-803 cells were incubated with formaldehyde for 10 min to obtain the DNA-protein crosslinks. Then, cell lysates were subjected to sonication to get the chromatin fragments and specific antibody or IgG as a control were used for immunoprecipitation. Later, qPCR analysis was employed to analyze the precipitated chromatin DNA.

### RNA pull-down and mass spectrometry

SNHG22 pull-down was performed by Magnetic RNA-protein Pull-down Kit (Thermo) according to the manufacturer’s protocol. Firstly, 5′Biotin-labeled oligonucleotide probe targeting SNHG22 was synthesized and purchased from KeyGEN (Jiangsu, China). The RNA was then bound to the beads to orient the RNA for protein binding. RNA-bound beads were incubated with nuclear extraction at 70 °C for 5 min. After RNA slowly cooled down to room temperature, Streptavidin Magnetic Beads were added and incubated at room temperature for 30 min. After washing away the unbound RNA, the beads were incubated with Elution buffer and the Supernatant was obtained for further mass spectrum and western blot. The identified proteins were listed in Supplementary Table 2.

### RNA-protein immunoprecipitation (RIP)

RIP™ RNA-Binding Protein Immunoprecipitation Kit (Millipore, USA) was used for detecting the binding between SNHG22 and EZH2 or SNHG22 and Ago2. Briefly, GC cell lysates were incubated with magnetic beads conjugated with indicated antibody or IgG as a negative control. Followed qRT-PCR analysis was performed to detect the enrichment of SNHG22.

### Animal studies

All animal experiments were proved by Qingdao University. Briefly, BGC823 cells that stably transfected with sh-ctrl or sh-SNHG22 were collected and suspended in PBS on ice. Twelve female BALB/c nude mice were divided into 2 groups randomly. 5 × 10^6^ cells were subcutaneously injected in the thigh root of each mouse. Tumor volumes were monitored every day after injection. At end, the mice were executed and tumors were harvested for further qRT-PCR, western blot and immunohistochemical staining (IHC) experiments.

### IHC

For IHC analysis, clinical tissues and subcutaneous tumors were fixed and embedded with paraffin wax. Tissues were cut into 4 mm slides. After deparaffinization and rehydration, slides were dipped into sodium citrate buffer for antigen retrieval. Then, slides were blocked with 1% BSA, followed by primary antibodies incubation overnight. Then, slides were incubated with secondary antibodies. The staining intensity was then quantitatively scored. 0 if 0% of tumor cells exhibited positive staining, 1 for 0 to 1% positive cells, 2 for 2% to 10% positive cells, 3 for 11% to 30% positive cells, 4 for 31% to 70% positive cells, and 5 for 71% to 100% positive cells. In addition, the staining intensity was scored on a scale of 0-3: 0, negative; 1, weak; 2, moderate; and 3, strong. Total score was got by adding the proportion and intensity scores.

### Statistical analysis

Data are presented as mean ± SEM and were analyzed using GraphPad 8.01. The expression difference of SNHG22 in clinical samples, cell lines or from TCGA dataset was analyzed using Student’s *t*-test. Overall survival (OS) was calculated using the Kaplan-Meier method. two-way ANOVA, and chi-square tests were used for comparison. Statistical significance was set at *p* < 0.05.

## Results

### SNHG22 is overexpressed in gastric cancer

**Fig. 1:**
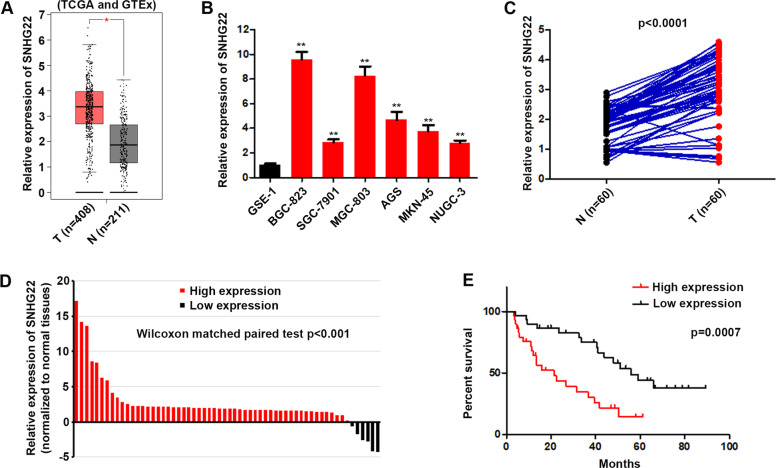
SNHG22 is overexpressed in gastric cancer. **A** Relative expression of SNHG22 in GC and adjacent normal samples were analyzed using TCGA dataset. **B** Relative expression of SNHG22 in GC cells were quantified by qRT-PCR. **C** Relative expression of SNHG22 in GC and adjacent normal samples were quantified by qRT-PCR. **D** Sixty GC samples were divided into two groups according to high and low SNHG22 expression. **E** Kaplan–Meier analysis of the association between SNHG22 high or low expression and the overall survival of GC patients. In all experiments, bars represent mean ± SD from three replicates (*n* = 3) (*P < 0.05, **P < 0.01).

The expression levels of SNHG22 were analyzed using TCGA dataset by GEPIA [13] and we found that SNHG22 was upregulated in gastric cancers compared with non-tumor tissues (Fig. 1A). In addition, we observed that the expression of SNHG22 was distinctly upregulated in all human GC cell lines compared with the GSE-1 cell line (Fig. 1B). Then, we analyzed the expression of SNHG22 in 60 paired GC tissues and normal tissues. qRT-PCR analysis showed that SNHG22 was upregulated in 44 GC tissues compared with the matched normal tissues (Fig. 1C and D). Furthermore, clinical samples were divided into two groups (high and low) according the median of SNHG22 expression level. The overall survival (OS) data showed that patients with high level of SNHG22 developed poor survival rate (Fig. 1E). Together, the up-regulated SNHG22 might contribute to the GC development.

### SNHG22 silencing inhibits GC cell proliferation and invasion

**Fig. 2:**
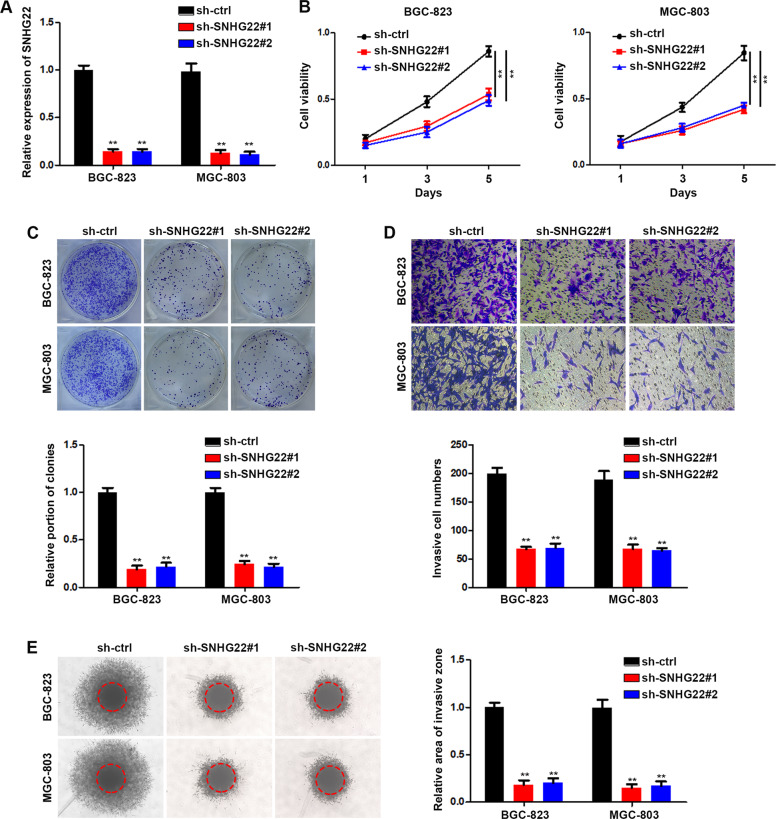
SNHG22 silencing inhibits GC cell proliferation and invasion. **A** Relative expression of SNHG22 in GC cells were quantified by qRT-PCR after transfection of sh-ctrl or sh-SNHG22. **B** The proliferation of transfected GC cells was evaluated using CCK-8 assay. **C** The proliferation of transfected GC cells was evaluated using colony formation assay. **D** The invasion of transfected GC cells was evaluated using transwell invasion assay. **E** The invasion of transfected GC cells was evaluated using 3D migration assay. In all experiments, bars represent mean ± SD from three replicates (*n* = 3) (*P < 0.05, **P < 0.01).

To explore the biologic function of SNHG22 in GC cells, we designed two shRNAs targeting the sequence of SNHG22 and qRT-PCR confirmed the silencing efficacy (Fig. 2A). CCK-8 and colony formation assays demonstrated that shRNAs transfection but not scrambled oligonucleotides significantly suppressed the growth and proliferation of GC cells (Fig. 2B and C). We also tested the effect of SNHG22 on the invasion of GC cells. Transwell assays indicated that SNHG22 silencing markedly suppressed normally strong invasive capacity of BGC-823 and MGC-803 cells (Fig. 2D). Meanwhile, SNHG22 silencing inhibited GC cells infiltration in a 3D collagen matrix (Fig. 2E). Taken together, these data indicated that SNHG22 depletion inhibited the GC progression in vitro.

### ELK4 binds to the promoter region of SNHG22 and upregulates its expression in GC cells

**Fig. 3:**
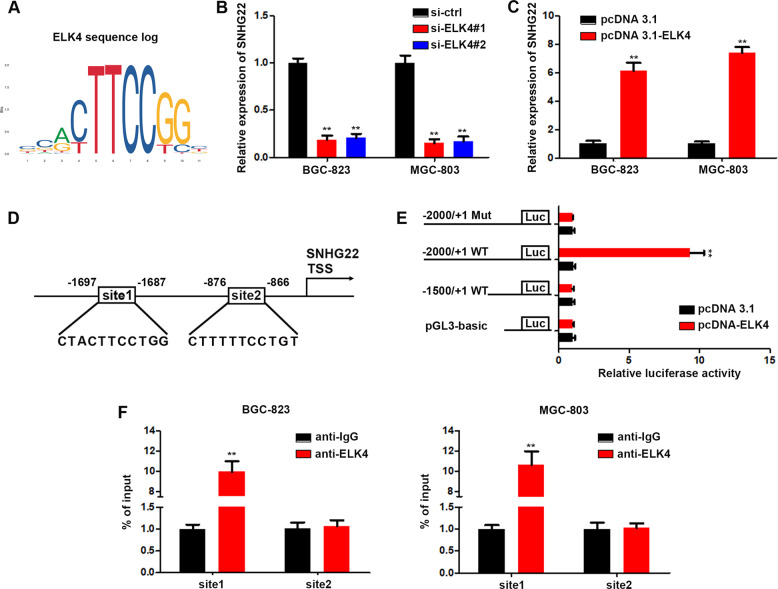
ELK4 binds to the promoter region of SNHG22 and upregulates its expression in GC cells. **A** Predicted biding sites of ELK4. **B** Relative expression of SNHG22 in GC cells were quantified by qPCR after transfection of si-ctrl or si-ELK4. **C** Relative expression of SNHG22 in GC cells were quantified by qPCR after transfection of pcDNA3.1 or pcDNA3.1-ELK4. **D** JASPAR algorithm predicted two biding sites (site1, site2) between ELK4 and SNHG22 promoter regions. **E** The luciferase reporter constructs carrying the human SNHG22 promoter region were transiently transfected into BGC-823 cells with either ELK4 or a mock control vector. **F** ELK4 bound to predicted binding sites in SNHG22 promoter was proved using ChIP assays. In all experiments, bars represent mean ± SD from three replicates (*n* = 3) (*P < 0.05, **P < 0.01).

We next sought to uncover the molecular mechanisms, which contributed to the upregulation of SNHG22 in GC cells. Accumulating researches have revealed that some lncRNAs transcription could be modulated by transcriptional factors (TFs) via directly binding [14, 15]. Therefore, we tend to explore if TFs can regulate SNHG22 expression. Using the JASPAR database (http://jaspar.binf.ku.dk/) [16], we found that ELK4 is a potential TF which promoting SNHG22 expression (Fig. 3A). In accordance with SNHG22, ELK4 expression levels were significantly higher in GCs compared with non-tumor tissues in TCGA dataset (Supplementary Fig. 1A). Next, we knocked down or overexpressed ELK4 in GC cells (Supplementary Fig. 1B and C). qRT-PCR results showed that SNHG22 level was positively correlated with ELK4 level (Fig. 3B and C). Furthermore, we found two possible binding sites (site 1 and site 2) of ELK4 in SNHG22 promoter regions (Fig. 3D). Then, we generated luciferase reporter constructs containing fragments of nucleotides −2000 to +1 of the human SNHG22 promoter region and examined their responsiveness to ELK4 (Fig. 3E). We found that ELK4 overexpression could not induce the activation of the reporter constructs containing the site 2 fragment, while the activity of reporter constructs containing both site 1 and site 2 fragments was significantly increased induced by ELK4 (Fig. 3E). In addition, we mutated site 1 and the reporter construct containing a mutant site 1 could not be activated by ELK4 overexpression(Fig. 3E). Meanwhile, we performed a chromatin immunoprecipitation (ChIP) assay using BGC-823 and MGC-803 cells. We found that site 1-containing DNA fragment, but not site 2 DNA fragment, of the SNHG22 gene was coprecipitated with ELK4, supporting the notion that ELK4 directly activates the SNHG22 promoter through binding the site 1 (Fig. 3F). Finally, we explored the impact of ELK4 on the malignancy development of GC cells. As shown in Supplementary Fig. 1D–G, ELK4 knockdown led to significant decrease of cell viability and invasion of GC cells. Together, our findings proved that ELK4 activated the transcription of SNHG22 in GC.

### SNHG22 interacts with EZH2 inducing accumulation of H3K27 trimethylation to multiple tumor suppressive genes

To further evaluate the molecular mechanism by which SNHG22 promotes GC progress, we firstly used FISH and subcellular fractionation to analyze its distribution. The results showed that SNHG22 was primarily observed in both nuclear and cytoplasm (Supplementary Fig. 2A and B). Hence, we tend to figure out the role of SNHG22 in different distribution separately.

**Fig. 4:**
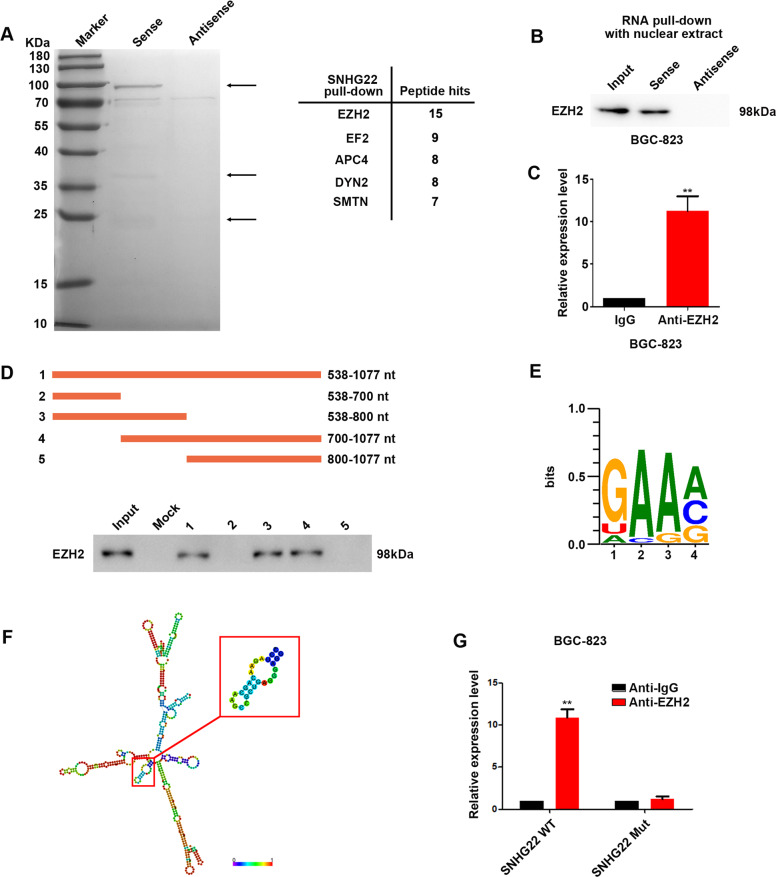
SNHG22 interacts with EZH2. **A** RNA pull-down of SNHG22 followed by mass spectrometry identification. The bands in frames were specifically precipitated by SNHG22, but not by antisense RNA. **B** SNHG22 pull-down followed by western blot exhibited the binding of SNHG22 to EZH2. **C** RIP assay showed the binding of SNHG22 to EZH2. **D** Serial deletions of SNHG22 were used in RNA pull-down assays to identify regions required for SNHG22 and EZH2 interaction. **E** POSTAR2 prediction of sequence motifs of EZH2 binding sites. **F** RNAalifold predicted the EZH2 binding stem-loop structures in SNHG22 (framed in red). **G** RIP assays performed after site-directed mutagenesis of 737–763 nt of SNHG22 in BGC-823 cells. In all experiments, bars represent mean ± SD from three replicates (*n* = 3) (*P < 0.05, **P < 0.01).

To identify the mechanism of nuclear SNHG22, biotinylated SNHG22 and antisense control were incubated with nuclear extraction. Mass spectrometry (MS) confirmed the specific band of SNHG22 pull-down products was enhancer of zeste homolog 2 (EZH2) (Fig. 4A). EZH2 is the functional enzymatic component of the Polycomb Repressive Complex 2 (PRC2), with a nuclear distribution in GC cells [17]. We confirmed the interaction of SNHG22 with EZH2 using RNA pull-down assay followed by western blotting (Fig. 4B and Supplementary Fig. 2C). Consistently, RNA immunoprecipitation (RIP) showed enrichment of SNHG22 by EZH2 (Fig. 4C and Supplementary Fig. 2D). These results validated the interaction between SNHG22 and EZH2. Moreover, serial deletion analysis validated that the 538–1077 nt region of SNHG22 was indispensable for direct interaction with EZH2 (Supplementary Fig. 2E). Sequence analysis by POSTAR2 (http://lulab.life.tsinghua.edu.cn/postar/) [18, 19] indicated a sequence motif and structural preference of the RBP binding site for EZH2 (Fig. 4E), which was located in the 737-763 nt region of SNHG22 and formed a stem-loop structure (Fig. 4F). RIP performed after mutagenesis of this stem-loop region revealed that it was critical to SNHG22 interaction with EZH2 (Fig. 4G and Supplemental Fig. 2F). These data suggest that SNHG22 interacts with EZH2 in nuclear.

**Fig. 5:**
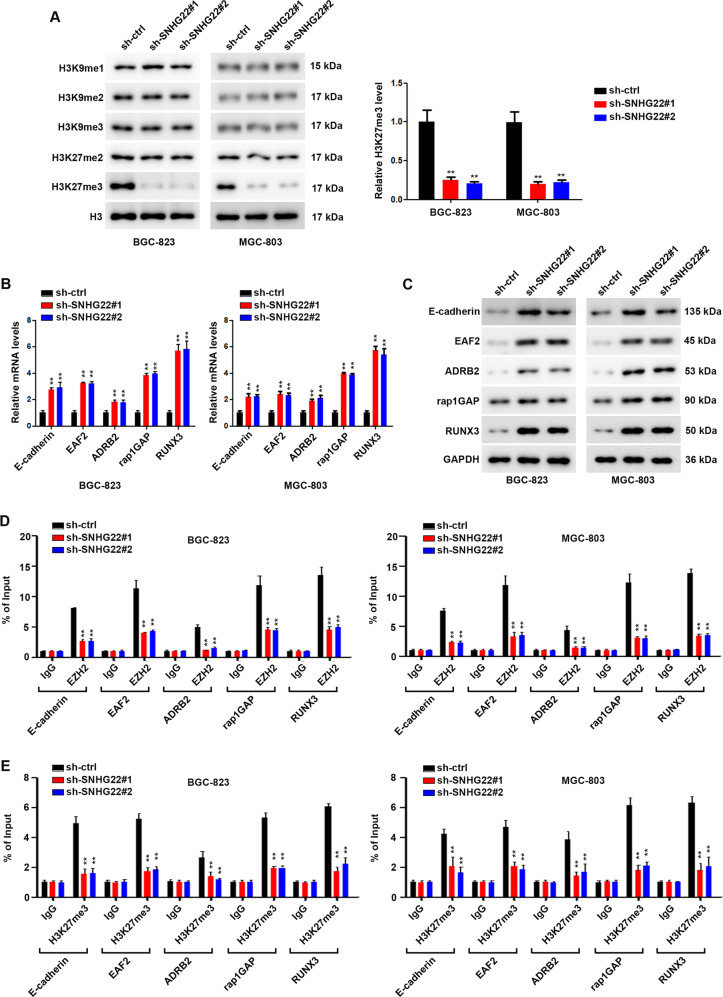
SNHG22 interacts with EZH2 inducing accumulation of H3K27 trimethylation to multiple tumor suppressive genes. **A** Relative expression of H3 methylation products in GC cells was analyzed using western blot after transfection of sh-ctrl or sh-SNHG22. **B** Relative expression of indicated genes in GC cells was analyzed using qPCR after transfection of sh-ctrl or sh-SNHG22. **C** Relative expression of indicated genes in GC cells was analyzed using western blot after transfection of sh-ctrl or sh-SNHG22. **D** EZH2 bound to predicted binding sites in indicated genes promoter was proved using ChIP assays. **E** H3K27me3 bound to predicted binding sites in indicated genes promoter was proved using ChIP assays. In all experiments, bars represent mean ± SD from three replicates (*n* = 3) (*P < 0.05, **P < 0.01).

As EZH2 is the enzymatic subunit of polycomb-repressive complex 2(PRC2). And PRC2 functions as a histone H3 lysine 27 (H3K27) methyltransferase to promotes transcriptional silencing via regulating chromatin structure. At the same time, histone methylation is involved in transcriptome reprogramming during GC development. Considering the interaction between SNHG22 and EZH2, we therefore tend to figure bout whether SNHG22 could regulate histone methylation. The results showed that SNHG22 deficiency in GC cells preferably decreased trimethylation at H3K27 instead of the levels of methylation at H3K9 (Fig. 5A), indicating a critical control mechanism of H3K27me3 by SNHG22. qPCR was performed to examine the mRNA levels of five common EZH2 target suppressive genes. The results demonstrated that the silence of SNHG22 remarkably increased the mRNA and protein levels in both BGC-823 and MGC-803 cells (Fig. 5B and C). Furthermore, ChIP assays revealed that SNHG22 knockdown remarkably decreased the binding of EZH2 to the promoter regions of these tumor suppressive genes (Fig. 5D and E).

Taken together, SNHG22 represses multiple suppressive genes by recruiting EZH2 to promote GC progression.

### SNHG22 promotes Notch1 expression through sponging miR-200c-3p

Using online prediction tools (SNP2 and starbase) [20, 21], we found four potential miRNAs which could interact with SNHG22 (Supplementary Fig. 3A). To figure out which one is the target of SNHG22, a dual luciferase reporter assay was performed and the results showed that miR-200c-3p significantly reduced the relative luciferase activity of the SNHG22 3’UTR (Supplementary Fig. 3B). Furthermore, we constructed mutant SNHG22 reporter plasmid (Supplementary Fig. 3C). The results of dual luciferase reporter assay showed that miR-200c-3p significantly suppressed the activity while the mutation of miR-200c-3p binding sites abolished this effect (Supplementary Fig. 3D). In addition, Ago2 based RIP assays confirmed the interaction between SNHG22 and miR-200c-3p mediated by Ago2 (Supplementary Fig. 3E). Next, we transfected GC cells with sh-SNHG22 or co-transfected sh-SNHG22 together with miR-200c-3p or anti-miR-200c-3p. In vitro experiments, including colony formation assays and 3D collagen matrix invasion assays, verified that knockdown of SNHG22 mediated inhibition of GC cell proliferation and invasion were partially rescued by co-transfection with anti-miR-200c-3p (Supplementary Fig. 3F and G). Together, these results suggested that carcinogenic effect of SNHG22 was partially mediated by sponging miR-200c-3p.

Next, using online prediction tools (DIANA TOOLS, miRDB and Targetscan) [22–24], we found 411 potential targets of miR-200c-3p (Supplementary Fig. 4A). GO analysis results indicated that these targets are involved in cell proliferation and invasion process (Supplementary Fig. 4B). Among these putative targets, we selected eight common genes as potential components of the SNHG22 and miR-200c-3p ceRNA network. qRT-PCR revealed that overexpression of miR-200c-3p significantly inhibited Notch1 expression in GC cells, while anti-miR-200c-3p exhibited the opposite effect (Supplementary Fig. 4C and D). Then, we constructed wild type and mutant Notch1 reporter plasmids (Supplementary Fig. 4E). The results of dual luciferase activity assays demonstrated that miR-200c-3p bound directly to the Notch1 3′-UTR region (Supplementary Fig. 4F). Overexpression of miR-200c-3p remarkably increased Notch1 expression, while knockdown of miR-200c-3p significantly reduced Notch1 expression (Supplementary Fig. 4G). These results suggested that Notch1 is a direct target of miR-200c-3p.

**Fig. 6:**
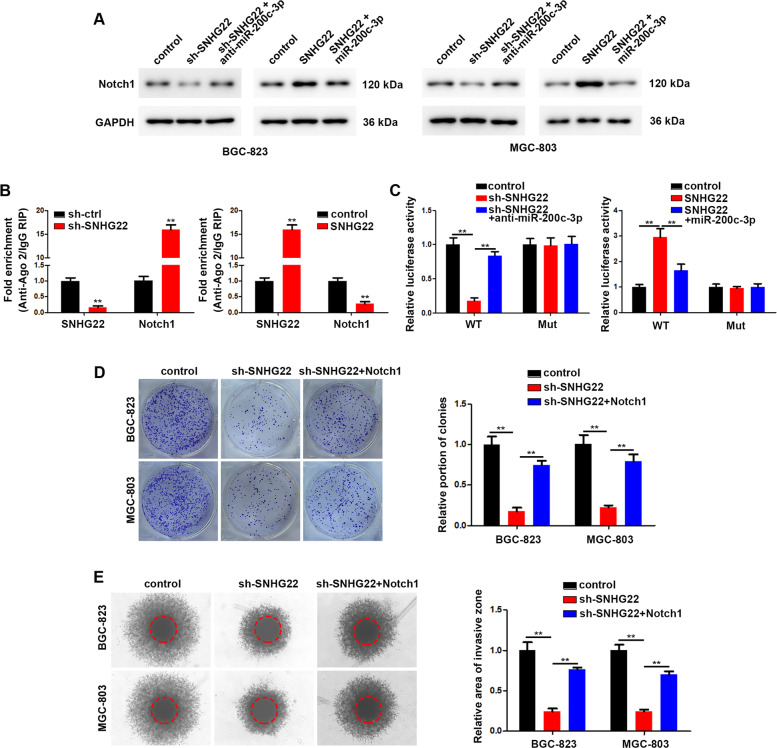
SNHG22 promotes Notch1 expression through sponging miR-200c-3p. **A** Relative expression of Notch1 in GC cells was analyzed using western blot after transfection. **B** RIP assay of the enrichment of Ago2 on SNHG22 and Notch1 transcripts relative to IgG in transfected GC cells. **C** Luciferase activity of reporters which contained wild-type or mt Notch1 3’UTR with indicated treatment in GC cells. **D** The proliferation of transfected GC cells was evaluated using colony formation assay. **E** The invasion of transfected GC cells was evaluated using 3D migration assay. In all experiments, bars represent mean ± SD from three replicates (*n* = 3) (*P < 0.05, **P < 0.01).

Notch1 expression was decreased upon SNHG22 knockdown but was rescued by anti-miR-200c-3p. Conversely, Notch1 expression was increased upon SNHG22 overexpression but was rescued by miR-200c-3p in GC cells (Fig. 6A). SNHG22 knockdown in GC cells elicited a marked increase in the recruitment of Ago2 to Notch1 transcripts, and overexpression of SNHG22 led to the increased enrichment of Ago2 transcripts bound to SNHG22 but decreased enrichment of Notch1 transcripts (Fig. 6B). The luciferase activity of Notch1 reporter was decreased upon SNHG22 knockdown and was rescued by miR-200c-3p sponge, while the luciferase activity of mutant reporter was unchanged (Fig. 6C). After identifying Notch1 as direct target of SNHG22, we next analyzed the functional role of Notch1 in GC progression. We observed that overexpression of Notch1 restored the proliferation and invasion in GC cells with knocked-down SNHG22 (Fig. 6D and E).

Collectively, these results demonstrated that SNHG22 functions as a molecular sponge for miR-200c-3p to facilitate the expression of Notch1 to promote the proliferation and invasion of GC cells.

### Knockdown of SNHG22 inhibits GC progression in vivo

**Fig. 7:**
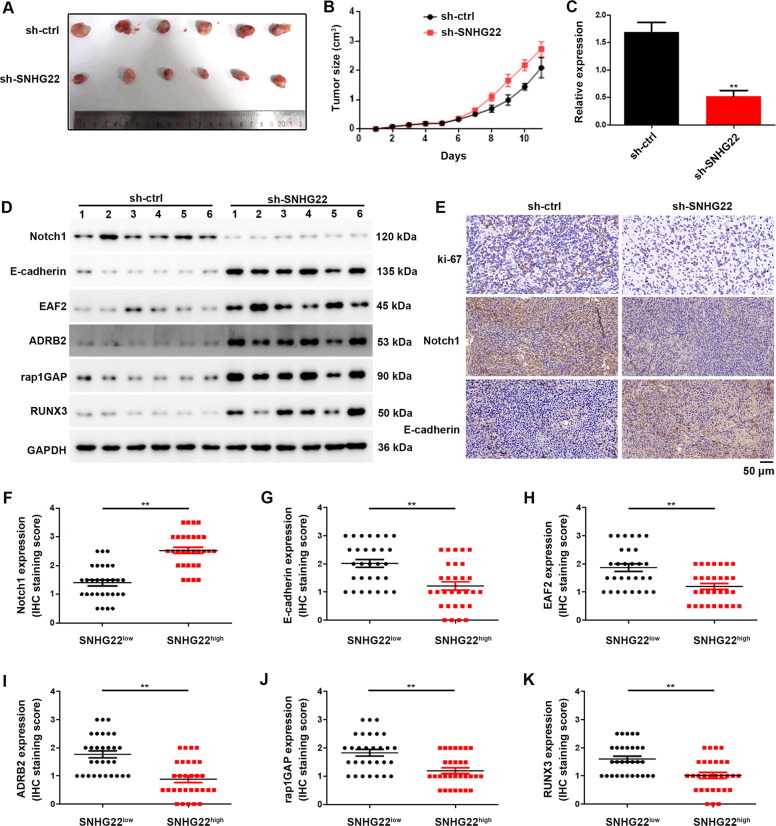
Knockdown of SNHG22 inhibits GC progression in vivo. **A** Subcutaneous tumors were separated and imaged at the endpoint of the experiment (*n* = 6). **B** Tumor growth curves of different subcutaneous tumor groups are shown. **C** Relative expression of SNHG22 in subcutaneous tumor tissues were quantified using qRT-PCR. **D** Relative expression of indicated genes in subcutaneous tumor tissues were quantified using western blot. **E** The expression levels of ki-67, Notch1 and E-cadherin in different groups of subcutaneous tumors were evaluated using IHC. **F**–**K** The inverse correlation between SNHG22 and Notch1, E-cadherin, EAF2, ADRB2, rap1GAP, RUNX3 expression in human GC tissues was analyzed using IHC.

To determine the impact of SNHG22 on tumorigenic capacities in vivo, a xenograft tumor model was constructed. SNHG22-stable-knockdown BGC-823 cells or control cells were subcutaneously injected into nude mice. We found that the tumors derived from the control group were markedly larger than tumors obtained from the SNHG22-stable-knockdown group (Fig. 7A and B). Additionally, tumors derived from the control group revealed higher SNHG22 levels (Fig. 7C) together with stronger Notch1 and lower E-cadherin, EAF2, ADRB2, rap1GAP and RUNX3 expression in tumors obtained from the SNHG22-stable-knockdown group (Fig. 7D and E). Together, these data indicated that SNHG22 promotes GC progression through elevating Notch1 and recruiting EZH2 to suppress tumor suppressive genes in vivo.

We next determined the expression levels of SNHG22 and the indicated genes in GC clinical samples. Consistent with the findings in mice, the higher Notch1 expression was observed in the SNHG22^high^ subtype GC clinical samples (Fig. 7F), and lower expression of E-cadherin, EAF2, ADRB2, rap1GAP and RUNX3 expression were ovserved in the SNHG22^high^ subtype GC clinical samples (Fig. 7G–K). These results confirmed the effect of SNHG22 on GC progression through regulating multiple genes.

## Discussion

Previous studies have revealed considerable roles for lncRNAs in the progression and development of human cancers [25, 26]. In gastric cancers, a lot of lncRNAs, like CRNDE [27], RNA1 [28], have been characterized and their functional mechanism have been reported as well. Recently, Shen et. al demonstrated that SNHG22 induces migration, invasion and angiogenesis of gastric cancer cells via miR-361-3p/HMGA1/Wnt axis [29]. However, the mechanism for high SNHG22 expression remains unknown. Meanwhile, deeper mechanism for SNHG22 regulating GC progression needs to be explored. In this study, we confirmed higher expression of SNHG22 in GC cells, and higher SNHG22 expression confers poor prognosis. In vitro and in vivo experiments supported the promotion of SNHG22 on proliferation and invasion of GC cells. These findings suggest that SNHG22 exerts an oncogenic function and play a key role in GC development.

ELK4 (SRF accessory factor-1, SAP-1) belongs to the TCF subfamily of the ETS domain transcription factors, and was described to have crucial roles in a lot of cancers [30]. ELK4 together with SRF is a significant regulator for many genes, for example c-fos and LAMB3 [31, 32]. In the present study, we found that ELK4 overexpression promotes proliferation and invasion of GC cells. Moreover, ELK4 can bind the promoter region of SNHG22 and regulate the expression level of SNHG22. Above all, this study found the regulatory mechanism of SNHG22 and indicated the important role of ELK4 on progression of GC.

LncRNAs were reported to regulate gene expression at the pre-transcriptional, transcriptional, and post-transcriptional levels [33]. At the transcriptional level, lncRNA regulates gene expression by modifying gene, histone and chromatin without changing the DNA sequences [34, 35]. In this study, we found that a large of SNHG22 was located at nuclear in GC cells, which conferred the potential of SNHG22 remodeling genes expression through EZH2. Using RIP and RNA pull-down assays, we found that SNHG22 directly binds EZH2 in GC cells. Further results of ChIP assays revealed that SNHG22 could recruit EZH2 to the promoter regions of multiple tumor suppressor genes (E-cadherin, EAF2, ADRB2, rap1GAP and RUNX3), and modulating H3K27me3 levels to repress their transcription. These findings indicate that lncRNA SNHG22 plays a crucial role in EZH2-mediated repression of tumor suppressor genes in GC cells.

At the post-transcriptional level, lncRNAs and miRNAs networks control the display of numbers of target genes. This ceRNA mechanisms emerged as imperative regulator for target genes [34, 36]. By online predictive tools and following RIP or luciferase activity assays, we discovered miR-200c-3p as the sponging miRNA of SNHG22. In addition, we found that Notch1 is the direct target of miR-200c-3p, and further results confirmed the regulation of SNHG22 on Notch1. Overall, our findings revealed the essential role of SNHG22/miR-200c-3p/Notch1 network on promoting GC progression.

In summary, our results establish highly expression of SNHG22 as a regulator of proliferation and invasion of GC cells. Our results further demonstrate that SNHG22 induces epigenetic silencing of E-cadherin, EAF2, ADRB2, rap1GAP and RUNX3 expression following binding with EZH2. Meanwhile, SNHG22 functions as a ceRNA to regulate Notch1 expression. These findings suggested that SNHG22 may serve as a potential therapeutic target and a prognostic biomarker in GC.

## Supplementary information

Supplementary Materials

## Data Availability

All relevant data within the scope of the paper are available from the corresponding author.
