# Supplementary material for: ELK4-mediated lncRNA SNHG22 promotes gastric cancer progression through interacting with EZH2 and regulating miR-200c-3p/Notch1 axis
Source: Cell Death Dis. 2021 Oct 18;12(11):957. doi: 10.1038/s41419-021-04228-z (PMC8523719; doi:10.1038/s41419-021-04228-z)
Supplement: Supplementary file 1 — Supplementary Materials [file 41419_2021_4228_MOESM1_ESM.docx]

**Supplementary Figures**

**
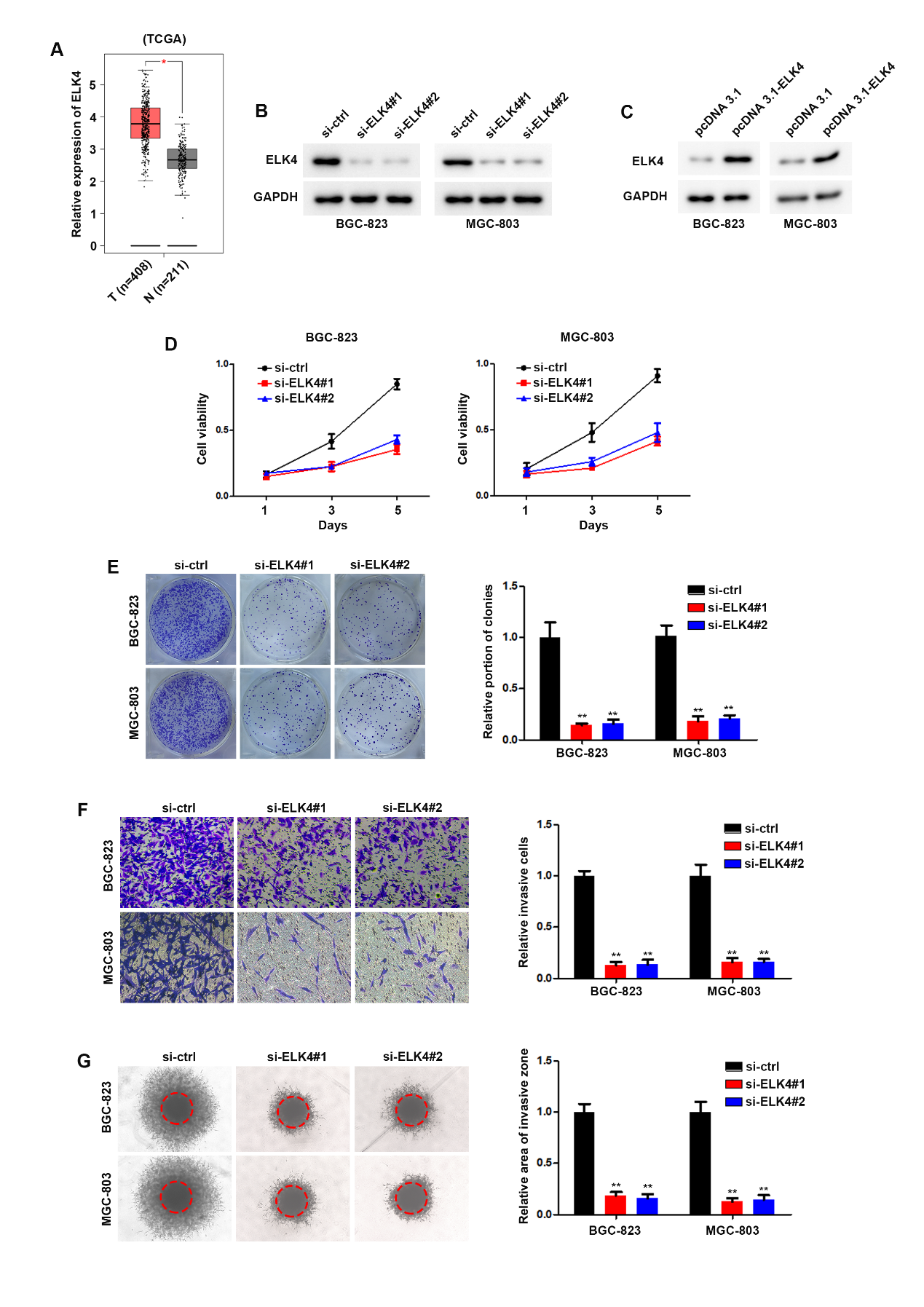
**

**Supplementary Fig. 1**

A, Relative expression of ELK4 in GC and adjacent normal samples were analyzed using TCGA dataset.

B, The expression of ELK4 in GC cells were measured by western blot after transfection of si-ctrl or si-ELK4.

C, The expression of ELK4 in GC cells were measured by western blot after transfection of pcDNA3.1 or pcDNA3.1-ELK4.

D, The proliferation of transfected GC cells was evaluated using CCK-8 assay.

E, The proliferation of transfected GC cells was evaluated using colony formation assay.

F, The invasion of transfected GC cells was evaluated using transwell invasion assay.

G, The invasion of transfected GC cells was evaluated using 3D migration assay.

In all experiments, bars represent mean ± SD from three replicates (n = 3). (**P*< .05, ***P*< .01.)


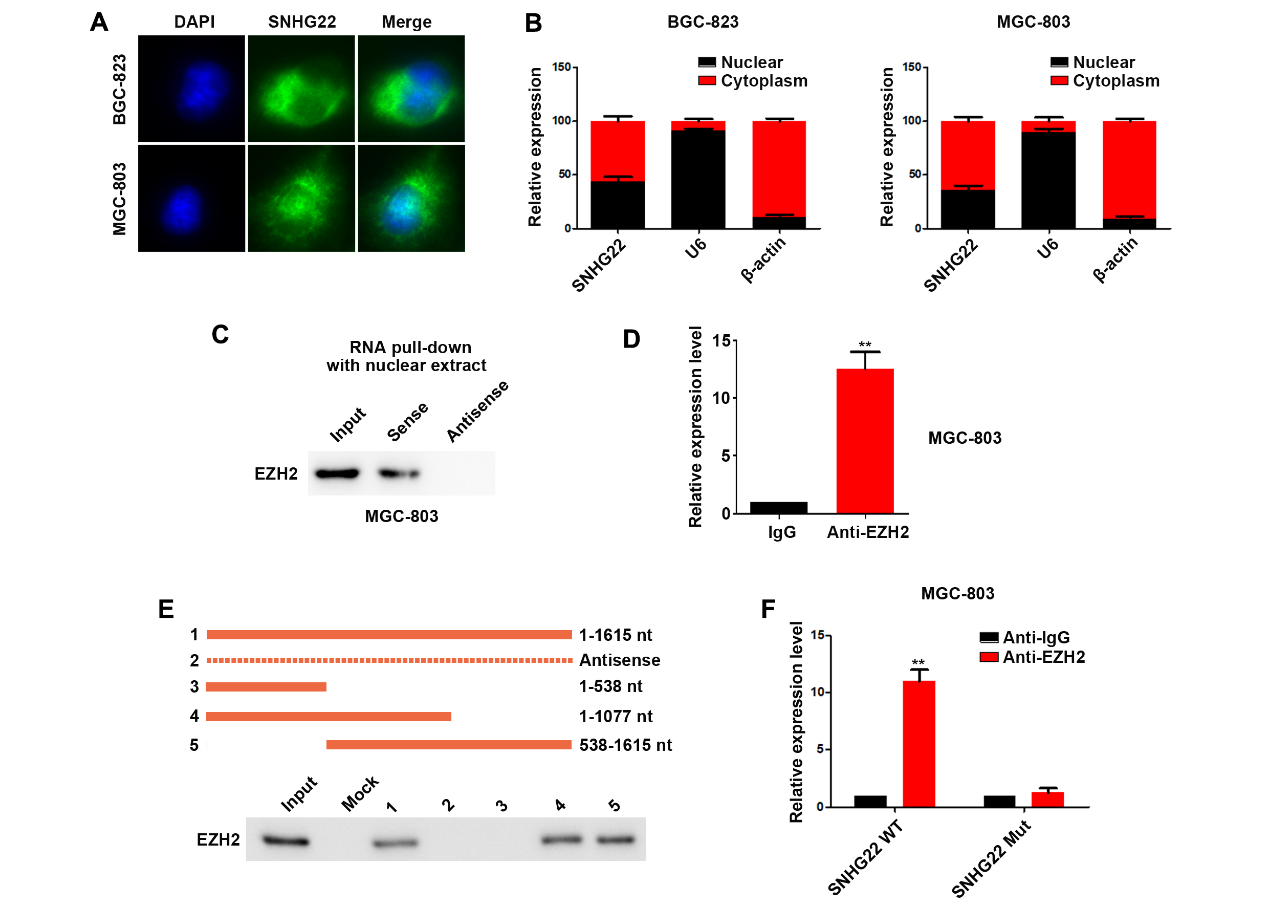


**Supplementary Fig. 2**

A, FISH analysis indicated subcellular location of SNHG22 in OS cells (green). Nuclei were stained by DAPI (blue).

B, Relative SNHG22 expression levels in nuclear and cytosolic fractions of GC cells were quantified by qRT-PCR.

C, SNHG22 pull-down followed by western blot exhibited the binding of SNHG22 to EZH2.

D, RIP assay showed the binding of SNHG22 to EZH2.

E, Serial deletions of SNHG22 were used in RNA pull-down assays to identify regions required for SNHG22 and EZH2 interaction.

F, RIP assays performed after site-directed mutagenesis of 737-763 nt of SNHG22 in MGC-803 cells.

In all experiments, bars represent mean ± SD from three replicates (n = 3). (**P*< .05, ***P*< .01.)


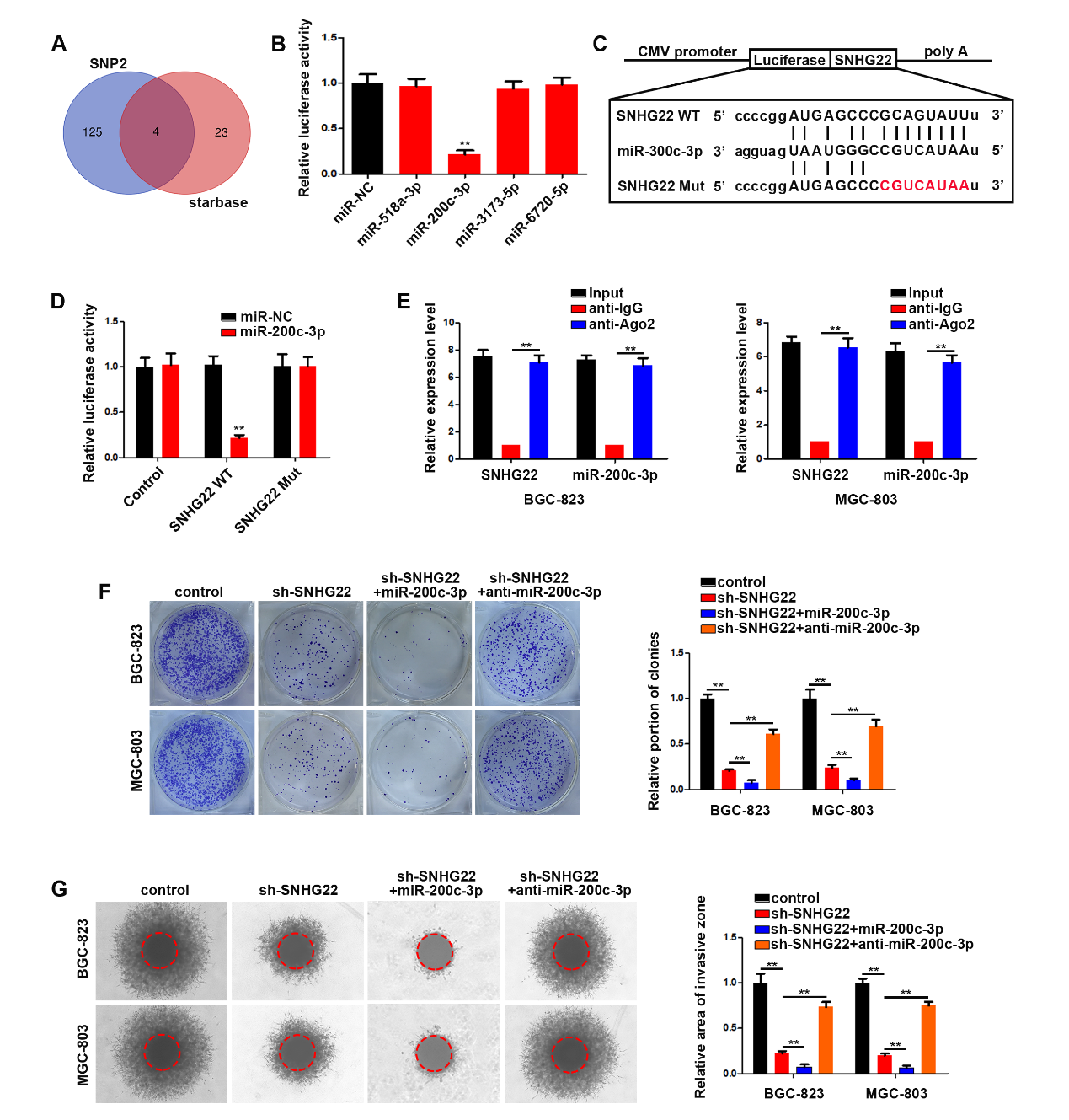


**Supplementary Fig. 3**

A, Schematic drawing of the screening procedure of candidate miRNAs.

B, The luciferase reporter plasmids carrying SNHG22 was co-transfected into HEK293T cells with 4 miRNA-coding plasmids.

C, Schematic representation of the miR-200c-3p binding sites in SNHG22 and the site mutagenesis.

D, The luciferase reporter plasmid carrying wild type (WT) or mutant (MUT) SNHG22 was co-transfected into GC cells with miR-200c-3p in parallel with an empty vector. Relative luciferase activity in GC cells were determined.

E, Ago2 RIP assay analysis of the enrichment of SNHG22 and miR-200C-3p pulled-down from the Ago2 protein in GC cells, and the expression levels of SNHG22 and miR-200c-3p were examined by qRT-PCR analysis.

F, The proliferation of GC cells after transfection with sh-SNHG22 or co-transfection with miR-200c-3p mimic or anti-miR-200c-3p was evaluated using colony formation assay.

G, The proliferation of GC cells after transfection with sh-SNHG22 or co-transfection with miR-200c-3p mimic or anti-miR-200c-3p was evaluated using 3D migration assay.

In all experiments, bars represent mean ± SD from three replicates (n = 3). (**P*< .05, ***P*< .01.)


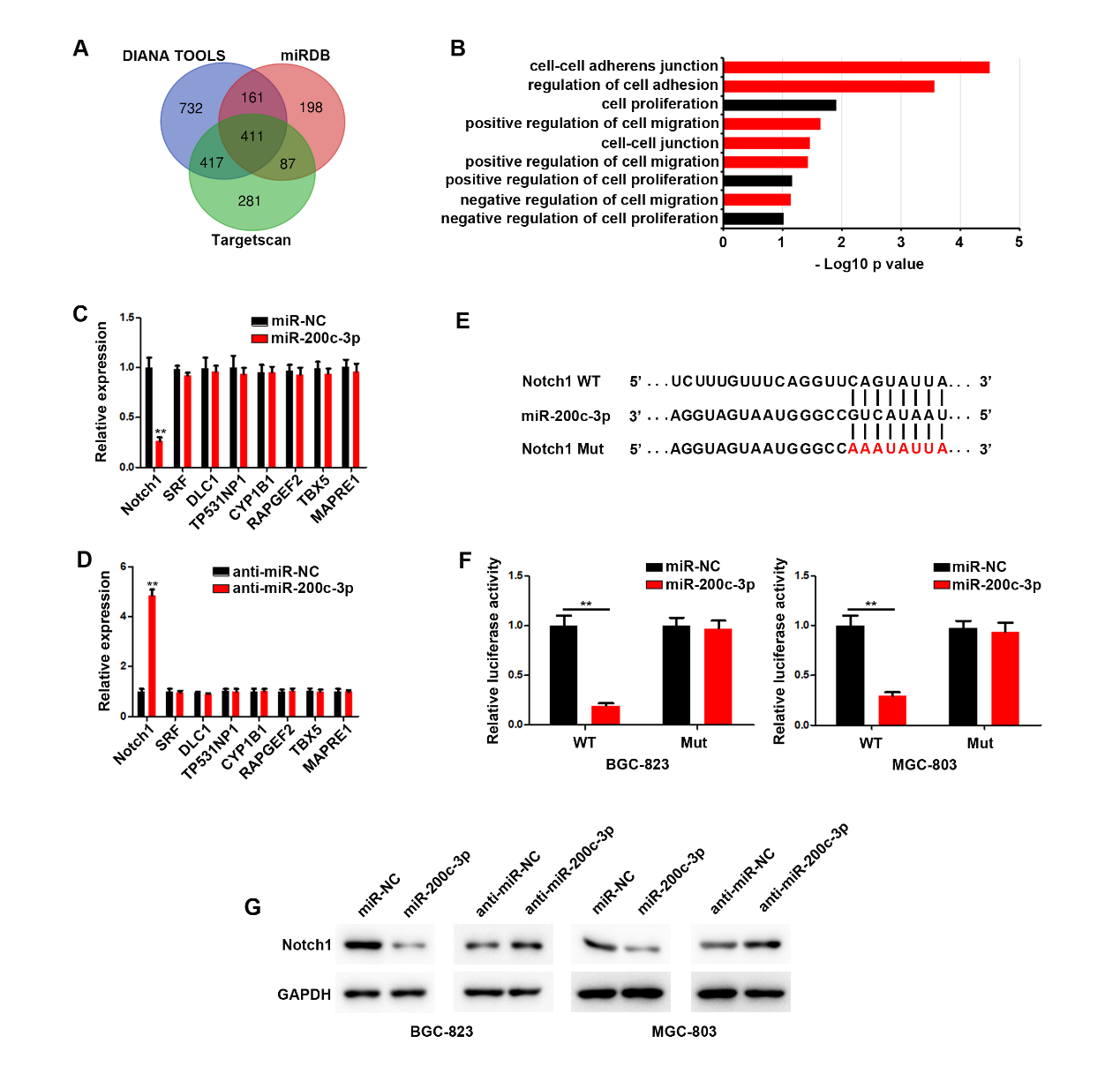


**Supplementary Fig. 4**

A, Schematic drawing of the candidate genes of miR-200c-3p using four prediction tools.

B, GO analysis of candidate targets of miR-200c-3p.

C, mRNA expression of candidates in GC cells transfected with miR-NC or miR-200c-3p mimic.

D, mRNA expression of candidates in GC cells transfected with anti-miR-NC or anti-miR-200c-3p.

E, Predicted miR-200c-3p target sequences in the 3′ UTRs of Notch1 genes.

F, Relative Notch1 reporter activities in GC cells co-transfected with miR-200c-3p and luciferase reporters.

G, Notch1 expression levels in GC cells transfected with miR-200c-3p mimic or anti-miR-200c-3p were quantified by western blot.

In all experiments, bars represent mean ± SD from three replicates (n = 3). (**P*< .05, ***P*< .01.)

**Supplementary Table 1**

|  | Forward | Reverse |
| --- | --- | --- |
| SNHG22 | AGGCGTGCACTACTGAGTTC | TTCGCCTCAGGGATTTGGAC |
| miR-200c-3p | CAGTGCGTGTCGTGGAGT | GGTAATACTGCCGGGTAAT |
| E-cadherin | GGTTTTCTACAGCATCACCG | GCTTCCCCATTTGATGACAC |
| EAF2 | CACCATGAATAGCGCAGCGGGATT | TCAGTCATCACTGTCACTTCCTGA |
| ADRB2 | GGTTATCGTCCTGGCCATCGTGTTTG | TGGTTCGTGAAGAAGTCACACCAAGTCTC |
| rap1GAP | GCCCACAACCAAGGTGAAG | CTGGACAAGATTAGGGAACTCG |
| RUNX3 | AGGCAATGACGAGAACTACTCC | CGAAGGTCGTTGAACCTGG |

**Supplementary Table 2**

| Protein ID | Protein name | Peptides | Unique peptides | Sequence coverage [%] | Unique sequence coverage [%] | Mol. weight [kDa] |
| --- | --- | --- | --- | --- | --- | --- |
| P23921 | EZH2 | 15 | 15 | 71 | 52.5 | 98.02 |
| P52732 | EF2 | 9 | 9 | 68.6 | 42.4 | 95.337 |
| E9PL69 | APC4 | 8 | 8 | 78.7 | 23.2 | 92.115 |
| Q9NR30 | DYN2 | 8 | 8 | 67.5 | 66.3 | 98.063 |
| P07900 | SMTN | 7 | 7 | 73.8 | 73.8 | 99.058 |
| P04264 | POLD3 | 7 | 7 | 66.9 | 56.2 | 100.28 |
| P08238 | RFC3 | 6 | 6 | 65.8 | 65.8 | 93.533 |
| P35908 | RFC4 | 6 | 6 | 54.1 | 54.1 | 99.996 |
| P13645 | PCNA | 5 | 5 | 54.6 | 54.6 | 91.88 |
| P55072 | COPS3 | 5 | 5 | 49.7 | 48.6 | 99.856 |
| Q08945 | COPS6 | 5 | 5 | 51.2 | 41.2 | 92.722 |
| Q14974 | COPS6 | 5 | 5 | 43.2 | 43.2 | 98.008 |
| H3BLV9 | RPA1 | 4 | 4 | 64.5 | 64.5 | 94.369 |
| O43143 | SSBP1 | 4 | 4 | 60.4 | 53.8 | 90.98 |
| P35527 | FEN1 | 4 | 4 | 47.8 | 45.8 | 98.636 |
| P56192 | VCP | 4 | 4 | 41.3 | 41.3 | 91.737 |
| O43167 | COPG1 | 4 | 4 | 45.8 | 45.8 | 97.717 |
| P02533 | XAB2 | 3 | 3 | 49.1 | 49.1 | 99.692 |
| P19338 | CALD1 | 3 | 3 | 37.3 | 26 | 93.23 |
| Q12906-5 | HMGB1 | 3 | 3 | 43.2 | 43.2 | 93.287 |
| Q7Z2T5 | RAD21 | 3 | 3 | 57.5 | 57.5 | 99.686 |
| E9PK47 | DDX1 | 3 | 3 | 44.3 | 1.2 | 96.793 |
| Q9NXF1-2 | MSH2 | 3 | 3 | 44.9 | 1.1 | 94.377 |
| A0A0U1RQT1 | KDM1A | 3 | 3 | 36.3 | 36.3 | 92.902 |
| B7Z6D5 | RPA2 | 3 | 3 | 57.4 | 57.4 | 99.057 |
| P13647 | X5D2M8 | 3 | 3 | 45.1 | 45.1 | 99.326 |
| Q9BQ39 | MRE11 | 3 | 3 | 45.7 | 45.7 | 92.48 |
| Q9Y4W2-2 | MA7D3 | 2 | 2 | 36.6 | 36.6 | 98.428 |
| O00139-2 | MTA1 | 2 | 2 | 41.7 | 41.7 | 97.552 |
| Q12788 | B4DVQ2 | 2 | 2 | 41.9 | 41.9 | 93.983 |
| Q14684 | XRCC1 | 2 | 2 | 31.5 | 30.3 | 91.495 |
| Q9UBF2-2 | MBD4 | 2 | 2 | 22.4 | 22.4 | 100.84 |
| O43395 | EP300 | 2 | 2 | 13.5 | 13.5 | 91.838 |
| Q9HCS7 | B3KXZ4 | 2 | 2 | 21.1 | 21.1 | 91.271 |
| Q9ULX6-2 | NUP93 | 2 | 2 | 20.4 | 20.4 | 93.487 |
| M0R3F1 | Q71SV8 | 2 | 2 | 16.7 | 16.7 | 99.705 |
| B1AHB1 | STT3B | 2 | 2 | 23.3 | 17.8 | 93.673 |
| E9PLT0 | SLFN5 | 2 | 2 | 16.1 | 16.1 | 101.05 |
| P05023-2 | LIG3 | 2 | 2 | 16.9 | 16.9 | 98.004 |
| Q92973-3 | APEX1 | 2 | 2 | 20 | 20 | 99.365 |
| Q9H3U1-2 | PARP1 | 2 | 2 | 19.6 | 19.6 | 93.233 |
| Q9UKV8-2 | PARP2 | 2 | 2 | 22.7 | 22.7 | 95.425 |
| H7C0R3 | POLR2G | 2 | 2 | 20.3 | 20.3 | 97.183 |
| H7BXI1 | PSMD2 | 2 | 2 | 22.9 | 22.9 | 100.2 |
| B8ZZS4 | POLR2H | 2 | 2 | 25.9 | 25.9 | 95.337 |
| P02765 | B3KY60 | 2 | 2 | 20.3 | 20.3 | 92.276 |
| P09874 | A8K492 | 2 | 2 | 20.2 | 14.8 | 101.14 |
| P14625 | SF3B2 | 2 | 2 | 12.9 | 12.9 | 100.23 |
| P23246-2 | B4DHJ4 | 1 | 1 | 21.2 | 21.2 | 99.277 |
| Q00839-2 | PRKDC | 1 | 1 | 15.2 | 15.2 | 98.096 |
| Q7Z353-2 | B3KPR5 | 1 | 1 | 20.6 | 18.6 | 96.08 |
| Q9UBT2 | Q2L6I2 | 1 | 1 | 41.6 | 41.6 | 91.679 |
| F1T0B3 | UBE2N | 1 | 1 | 17.6 | 17.6 | 99.31 |
| A0A0A0MR66 | MCM6 | 1 | 1 | 17.8 | 17.8 | 92.888 |
| A0A0B4J1W3 | COPS3 | 1 | 1 | 17.6 | 17.6 | 92.115 |
| C9J6P4 | COPS6 | 1 | 1 | 12.7 | 12.7 | 100.39 |
| E9PFK5 | COPS2 | 1 | 1 | 24.5 | 24.5 | 92.249 |
| E9PK09 | GPS1 | 1 | 1 | 15.5 | 15.5 | 93.296 |
| G5EA36 | F4ZW66 | 1 | 1 | 16.8 | 16.8 | 95.777 |
| O43264-2 | PML | 1 | 1 | 19.9 | 19.9 | 97.55 |
| P11387 | AF1L2 | 1 | 1 | 20 | 20 | 91.299 |
| P13010 | CHEK1 | 1 | 1 | 11.6 | 11.6 | 91.084 |
| P81605 | RPS27L | 1 | 1 | 12.2 | 12.2 | 92.339 |
| Q15393 | DLG1 | 1 | 1 | 15.8 | 15.8 | 100.45 |
| Q86TB9-2 | TF3C4 | 1 | 1 | 7.4 | 7.4 | 91.981 |
| Q9GZL7 | SNUT1 | 1 | 1 | 8.1 | 8.1 | 90.254 |
| Q9Y5V3 | SRRT | 1 | 1 | 8.3 | 8.3 | 100.67 |
| A0A024RAC6 | TF3C2 | 1 | 1 | 11.6 | 11.6 | 100.68 |
| A0A087WV90 | Q8NEH0 | 1 | 1 | 8.3 | 8.3 | 92.155 |
| A0A087WWD6 | RBM25 | 1 | 1 | 7.1 | 7.1 | 100.18 |
| A0A087X0Q4 | Q6NT15 | 1 | 1 | 6.3 | 6.3 | 100.94 |
| A0A075B730 | EXOSX | 1 | 1 | 6.6 | 6.6 | 100.83 |
| B7ZBT8 | MATR3 | 1 | 1 | 21.7 | 21.7 | 94.622 |
| Q8WY19 | TOP1 | 1 | 1 | 6.3 | 6.3 | 90.725 |
| H7BXY1 | B2R5U1 | 1 | 1 | 4.9 | 4.1 | 99.67 |
